# The Design Help Desk: A collaborative approach to design education for scientists and engineers

**DOI:** 10.1371/journal.pone.0212501

**Published:** 2019-05-01

**Authors:** Timothy O’Mahony, Jason Petz, Jonathan Cook, Karen Cheng, Marco Rolandi

**Affiliations:** 1 Institute for Connecting Neuroscience with Teaching and Learning (iCNtl), Seattle, WA, United States of America; 2 LIFE (Learning in Informal and Formal Environments) Center, College of Education, University of Washington, Seattle, WA, United States of America; 3 Division of Design, School of Art + Art History + Design, University of Washington, Seattle, WA, United States of America; 4 Department of Electrical and Computer Engineering, University of California, Santa Cruz, CA, United States of America; University of Idaho, UNITED STATES

## Abstract

Visual design, learning sciences, and nanotechnology may be strange bedfellows; yet, as this paper highlights, peer interaction between a designer and a scientist is an effective method for helping scientists acquire visual design skills. We describe our findings from observing twelve sessions at the Design Help Desk, a tutoring center at the University of Washington. At each session, a scientist (who is expert in his own domain but a novice in design) consulted a designer (who is expert in design but a novice in science) in order to receive advice and guidance on how to improve a scientific visualization. At the Design Help Desk, this pairing consistently produced a momentary disequilibrium in the scientist’s thought process: a disequilibrium that led to agency (where the scientist gained ownership of his/her own learning) and conceptual change in the scientist’s understanding of visual design. Scientists who visited the Design Help Desk were satisfied with their experience, and their published work demonstrated an improved ability to visually communicate research findings—a skill critical to the advancement of science. To our knowledge, the Design Help Desk is a unique effort to educate scientists in visual design; we are not aware of any other design-advice/tutoring centers at public or private universities in the United States or abroad.

## Introduction

Graduate students in science and engineering typically experience in-depth, rigorous training on how to conduct cutting-edge research. However, they receive far less education on how to effectively communicate this research—especially in the area of scientific visualization. Even though graduate students are expected to produce high-quality figures for scientific papers[1–3], posters and presentations, their studies rarely include coursework in visual design or visualization techniques. Instead, students learn in an ad-hoc fashion from peers and advisors, acquiring visual skills without a clear framework[4]. This lack of knowledge in visual design is unfortunate, because visualization can be a powerful tool for scientific discovery, as well as a compelling vehicle for sharing information[5]. By creating visual representations, scientists can explore speculative concepts, deconstruct complex relationships, and discover patterns and key features in their data. In addition, by externalizing their thinking in visualizations that can be shared with others, scientists facilitate communication with other scientists and the general public. There are numerous examples of scientific visualizations that have functioned as powerful and inspiring public revelations—ranging from Galileo’s drawings of the Earth’s moon,[6] to Watson and Crick’s double-helix model of the DNA molecule [7].

To address the gap in visual learning, a number of guides to designing effective visuals have been written for scientists and engineers.[8, 9] Many of these guides offer valuable frameworks for thinking about visual communication design and include useful comparisons of effective and ineffective scientific graphics. However, as many educators have discovered[10], simply creating the solution in a body of material and having it available will not necessarily cause the learner (in this case the scientist) to understand the content and the application of the processes prescribed therein.[11] Even though these texts are intended to be accessible to non-designers, they can be overwhelming to novices, who may have difficulty determining which lessons apply to their own work. In design, there are few black-and-white rules and rubrics that can be applied in a straightforward manner; most design decisions involve the subjective consideration of competing attributes.

At the University of Washington, we sought to make design knowledge more accessible to scientists and engineers by creating a visual design tutoring center called the Design Help Desk. At the Design Help Desk, science and engineering graduate students can consult with a design graduate student or senior design undergraduate to receive visual advice and guidance on their figures, presentations, posters—any scientific visualization or graphic.

In operating the Design Help Desk, we became interested in understanding what kind of learning was taking place between scientists and designers. Were scientists gaining skills and knowledge, or were they simply following the specific directions of the design consultant?

If design-science consultations are indeed effective in helping scientists and engineers acquire visual design skills, such a model would be relatively simple to replicate at universities world-wide. Unlike efforts to incorporate visual design coursework into the curriculum of a STEM degree program, peer-tutoring sessions can be organized with modest effort and funding. Furthermore, peer tutoring is time-efficient (in comparison to enrolling in a semester-long visual design course) and provides scientists with targeted visual instruction on a critical issue at hand.

## Materials and methods

### Subjects

The Internal Review Board (IRB) of the University of Washington granted permission for this study. We observed twelve subjects who voluntarily came to the Design Help Desk for advice on how to improve a visual figure that they had previously created in the course of their research. Participants learned about the Design Help Desk from a number of sources including: (i) advertisements placed on notice boards targeting University of Washington graduate students in science and engineering (see Supporting Information); (ii) classroom announcements by faculty and staff teaching in science and engineering; and (iii) word of mouth across campus as students discovered the availability of a tutoring center offering free help in designing visual figures.

Subjects included both graduate students and post-doctoral researchers studying science and engineering at the University of Washington (specific disciplines included the physical sciences, theoretical sciences, and nanoscience). There were an equal number of females to males, with ages ranging from mid-twenties through mid-fifties. The participants came from diverse backgrounds reflecting the general population of the University of Washington.

Subjects scheduled their 30-minute Design Help Desk appointment online. When subjects arrived at the Design Help Desk, they were reminded that their interaction could be part of a research study and were asked if they wanted to participate. Subjects were informed of their rights and told that if they preferred not to take part in the study that it would have no impact on whether or not the design consultant would help them (in fact, the designer never knew if a participant was in the study or not). When the subject agreed to be part of the study, they signed the research consent form. As it happened, all participants opted to be part of the study.

We sought and received Internal Review Board permission for this study. All necessary precautions were taken to preserve participant anonymity. Express written permission was sought and granted when pictorial images and verbal utterances of participants were used in associated publications and reports. Individuals in this manuscript have given written informed consent to publish their case details (as outlined in the PLOS consent form).

### Data collection

**Fig 1:**
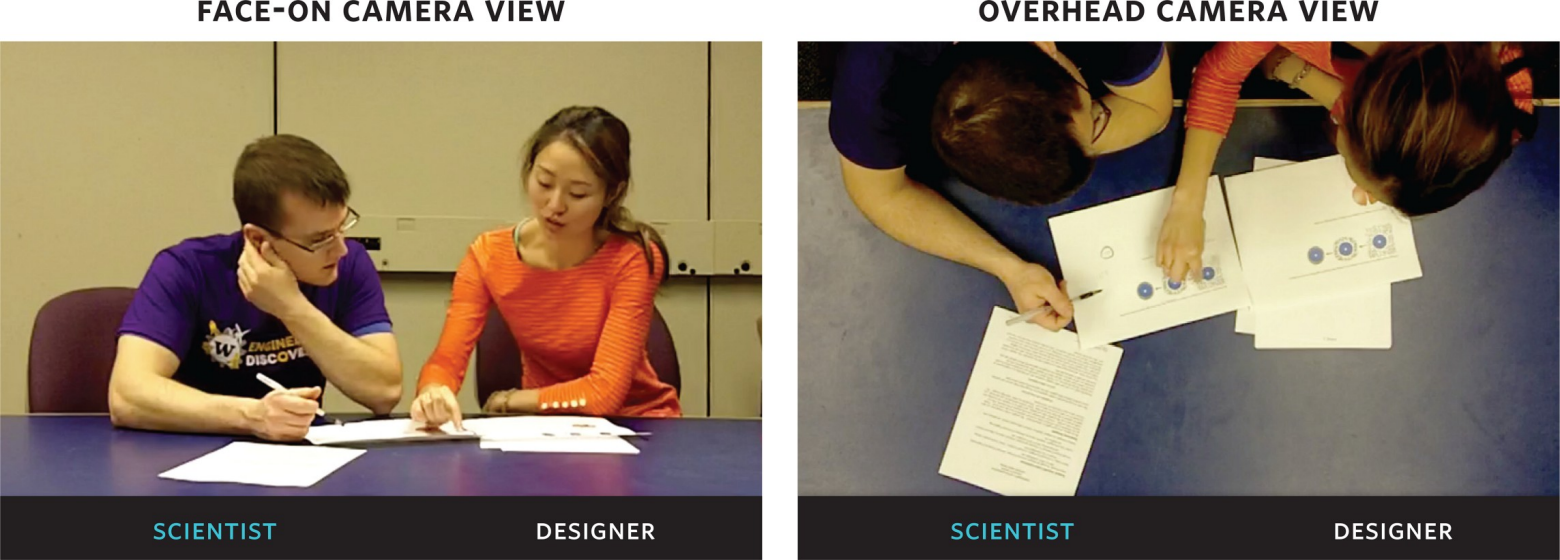
Camera setup at the design help desk. At the Design Help Desk the scientist was always seated on the left and the designer on the right.The image on the left shows the “Face-On View” which captures the frontal expressions, gestures and body language of both participants. The image on the right shows the “Overhead Desk View” which captures the gestures of both participants on the desktop, including interactions with the graphic printouts on the desk.

We collected a mix of written and audio/video material. First, we asked each participant to complete a written demographic survey. Then, we recorded all sessions of the Design Help Desk with two video cameras. The camera setup (as shown in Fig 1) was always the same. One video camera captured the frontal expressions, body language and gestures of both participants as they sat at the same desk facing forward directly into the camera. The designer was always seated on the right, and scientist was always seated on the left. The second video camera was placed over the desk, suspended from the ceiling. This second camera captured what happened on the desktop, including the hand positions of both participants with respect to the desk and any paperwork on the desk. Both cameras were turned on before the session began and were not turned off until after the scientist left the room.

After each DHD session, the scientist received an email inviting them to complete a satisfaction survey regarding their experience. This survey (Appendix B) also collected general demographic information, including academic background and previous experience/education in visual design.

To gauge the overall impact of the Design Help Desk, we contacted participants after their session to ask if they had made changes to their graphics following the consultation. We also asked if their graphic was subsequently published (for example, in paper, in a poster presentation, thesis, etc.) If we were unable to reach the participant, we conducted a literature and online search to determine if the graphic brought to the DHD had been published in a paper or other venue.

### Data analysis

We analyzed our video data using Interaction Analysis, as described by Jordan and Henderson.[12] Interaction analysis is an interdisciplinary method that uses empirical evidence to examine the interactions between human beings with each other, and with objects in their environment. Specifically, at the Design Help Desk, we examined both verbal and nonverbal interactions (as captured using the two-camera setup described above) to identify how issues relating to the scientist’s graphics unfolded and were resolved at the Design Help Desk.

First, each videotaped Design Help Desk session was transcribed (our transcription convention is detailed in Appendix C). Following the work of Goodwin [13] transcriptions included annotations for nonverbal communication such as changes in body position, gaze, and gesture.

Next, the videotapes for each session were played in front of a four-member working group. Each member of the group had been trained in interaction analysis methods and techniques. As the videos played, members marked categorical items of interest (e.g., conflict, agreement, collaboration, etc.) and halted playback for discussion of each marked incident. The individual stopping the video referenced specific verbal and/or non-verbal behavior to convince others of a particular claim or assertion about the apparent mental state or intention as evidenced in the interaction of the participants. Disagreement is common in this process, and in this case, was resolved through discussion and consensus.

**Fig 2:**
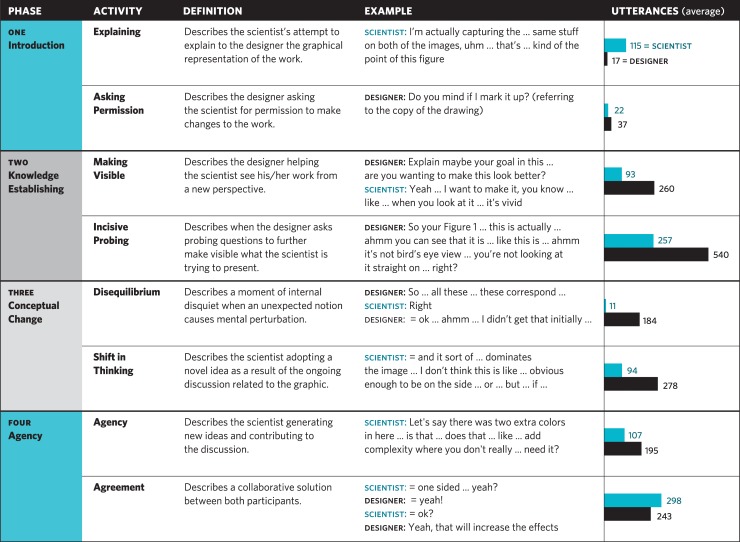
Cognitive interaction coding. Analysis of twelve Design Help Desk sessions identifies specific phases in the interactions between the designer and scientist. Two main activities occur during each phase. The last column shows the average number of utterances during each of these phases. Blue denotes utterances from the scientist, while black is used for the designer. The first and last phases are dominated by the scientist. The second and third phases have more utterances from the designer. Phases vary in length; Disequilibrium is relatively short, while the longest phase is Knowledge Establishing.

Based on the collaborative viewing and discussion process described above, the working group identified a series of four phases that occur in a consistent sequence during Design Help Desk sessions (see Fig 2 *Cognitive Interaction Coding*). Each phase includes two categories of activities/ interactions.

Following the efforts of the four-person working group, two members of the group then used the transcripts and accompanying video recordings to independently identify and code the agreed-upon stages and interactions in each of the twelve Design Help Desk sessions. Identification and selection of these interactional episodes correlated at ≥ .80, and interrater reliability among researchers was 0.95. Interrater Reliability was computed using Cronbach’s A, which may be thought of as the average correlation among the research team.[14] Again, disagreements were resolved through discussion and consensus.

As shown in the last column of Fig 2, we also measured the number and length of turn-taking utterances by each participant during each Design Help Desk session. We defined an utterance as any stretch of talk by one person, before and after which there is silence by that person. Using this definition, an utterance does not need to be grammatically correct. A participant who says “Well, I umm…” has spoken an utterance that is just as valid as a participant who says… “Well, I am not convinced.” As noted by Wooffitt, all aspects of interaction—even fillers (such as umm, huh, ah. h… etc.), repeated phrases and idiosyncratic words (e.g., like, we..ll… etc.)—are valid utterances that may be significant in illuminating a speaker’s mental state[15]

In order to process the large quantity of discourse generated by twelve half-hour sessions, we used the visualization tool “Monologger” created by Tad Hirsch and Jonathan Cook at the University of Washington[16] to carry out moment-to-moment video analyses of the interaction between the scientist and the designer. Monologger captures and visualizes content logs of conversations as bar charts, therefore ‘making visible’ key aspects of participant interaction, such as the number of utterances, overall speaking time, turn-taking, and over-talk. These aspects of conversation have proved valuable in understanding how participants verbally (and non-verbally) exchange ideas in conversation under specific communication situations[17].

## Results

Our video analysis shows that the Design Help Desk created a learning opportunity for scientists, with disequilibrium appearing to be the catalyst. According to Graesser[18], complex learning occurs when there is a discrepancy between an immediate situation and a person’s prior knowledge, skills and strategies—in this case, the scientist’s uncertainty regarding how to modify a visual to overcome deficits. In this scenario, the scientist experiences a gap in his/her knowledge—an impasse that causes confusion and frustration—what Piaget refers to as cognitive disequilibrium.[19] Piaget’s theories have been verified in many research studies using behavioral and empirical testing in learning settings as diverse as neuroscience laboratories,[20] classrooms,[21] and workplaces.[22]

Disequilibrium appears to be key. It is related to categories of information (Piaget's schema), which reside in a person’s executive function region (prefrontal cortex) and are central to consolidating learning through complex neural networks.[23] These networks become activated when an individual is prompted to re-evaluate prior knowledge and possibly assimilate it with new learning in the process of solving problems or creating new products.[24] This entails a transfer function[25, 26] that further promotes network activation with resulting neuroplasticity[27] to construct long-term memory[28] and deep understanding.[29, 30] Willis asserts that without these kinds of opportunities for strengthening, any memories learned by traditional methods (e.g., rote) are simply pruned away from disuse. Central to the learning that appears to be emergent in this interactive space is the notion of metacognition[31] that leads to agency[32] and personal fulfillment.[33]

To better understand the concept of disequilibrium, we review the phases of the Design Help Desk, as shown in Fig 2, above. In Phase One—*Introduction*—the designer (as the more experienced participant) begins the Design Help Desk session (See Appendix C, Consultant Script) by laying the groundwork for a proactive collaboration. However, the scientist dominates the conversation with roughly seven times more utterances than the designer (115 vs. 17), since it is his/her graphic that needs to be explained in context. We call this activity *Explaining*, since the scientist is describing and clarifying his graphic to the designer. During this time, the designer’s responses are generally reinforcing statements that demonstrate empathy—often simply listening and, when appropriate, repeating back to the scientist what was just stated—a feature of pedagogy that establishes safety, agency, and collaboration.[34] For example:

**Designer:** So (0.2) before we begin (0.1) do you wanna just kinda give me an overview of what they're (0.2) they're [the figures] saying? ((laughs))
**Scientist:** Sure
**Designer:** What they're about?
**Scientist:** Sure (0.1) uhm (0.2) so (0.2) the (0.1) these are both locations on Mars (0.1) which is what I study ((laughs))
**Designer:** OK (0.2) good ((nodding))
**Scientist:** Uhm (0.2) and (hhh) so the reason that I'm doing the study that I'm (0.3) these figures are for (0.2) is because (0.2) uh (0.2) my advisor actually found (0.2) um (0.2) quartz at these locations (0.3) which is rare on Mars (0.3) so, (0.2) quartz is all over the Earth (0.2) but it's not on Mars anywhere except this location
**Designer:** Huh
**Scientist:** We're trying to figure out why (Hhh)

The *Introduction* phase generally ends when the designer asks if they can make changes to the work in question—when he or she *Asks Permission* to modify elements of the visual under discussion. For example, the designer may ask the scientist, “Do you mind if I mark it up?” (referring to the paper copy of the visual graphic they are jointly examining).

In Phase Two—*Knowledge Establishing*—both participants ease deeper into understanding the specifics of the graphic positioned on the desk between them—the object of joint visual attention. The designer asks *Incisive Probing* questions to try and *Make Visible* what the scientist is trying to present. For example, in this excerpt, the designer needs to understand the intention of the scientist in his rendition of double lines to show tectonic activity after earthquake events:

**Scientist**: There are two significant earthquake events that could potentially affect Puget Sound (0.4) one of them was (0.2) uh an earthquake on the (0.3) what's called the Seattle fault? and that was about (0.3) um (0.1) 1100 years ago =
**Designer**: = Is that indicated by these two lines?
**Scientist**: That is indicated by this (0.2) this gray line here =
**Designer**: = Yeah? that gray line?

By digging deeper with questions, the designer is encouraging the scientist to explain his reasons for creating the existing graphic—and setting the groundwork for possibly considering a new visual design concept. At the same time, the designer is also monitoring the potential for the scientist to become protective and/or reactive. The designer is skilled in interpreting reactive states and is prepared to advance or relax the approach (depending on perceived resistance) so that the session can proceed productively.

During this *Knowledge Establishing* phase, the scientist typically takes a ‘follower’ role, and the ratio of utterances reflects this, with the Designer speaking roughly twice as much as the Scientist (260 vs. 93). For example, in the following excerpt, the designer has the dominant speaking role:

**Designer:** I would say that (0.2) uhm (0.2) yeah (hhh) I (0.1) I (0.2) the- these definitely get lost
**Scientist:** Mmhmm
**Designer:** I mean I saw them up here in the blue, (0.2) and then green (0.2) I mean it also is the print-out (0.2) like you mentioned
**Scientist:** Mmhmm
**Designer**: Um (0.3) I think calling out stuff is fine (0.2) I mean, = (0.3) what I'm seeing the most (0.3) and (0.2) and where you're gonna see the most is the contrast
**Scientist:** Mmhmm
**Designer:** And so right now the contrast that I (0.3) it's all these lakes
**Scientist:** Mmhmm
**Designer:** And that's sort of (Hhh) giving me a really strong indication of land mass
**Scientist:** Mmhmm
**Designer:** But not necessar- (0.2) then I lose everything else
**Scientist:** Mmhmm

Phase Three—*Conceptual Change*—is dominated by two elements that are a critical turning point in the overall reaction of the scientist to the new information and to the realization that change was needed. The first is the moment of *Disequilibrium*—the Piagetian learning concept[35] that we discussed previously as a requirement for conceptual change.

[36] The second is a *Shift in Thinking*—a moment of acceptance[37] when the scientist jumps back into the fray by generating new ideas[38] and contributing elements that advance the project with the designer.

**Fig 3:**
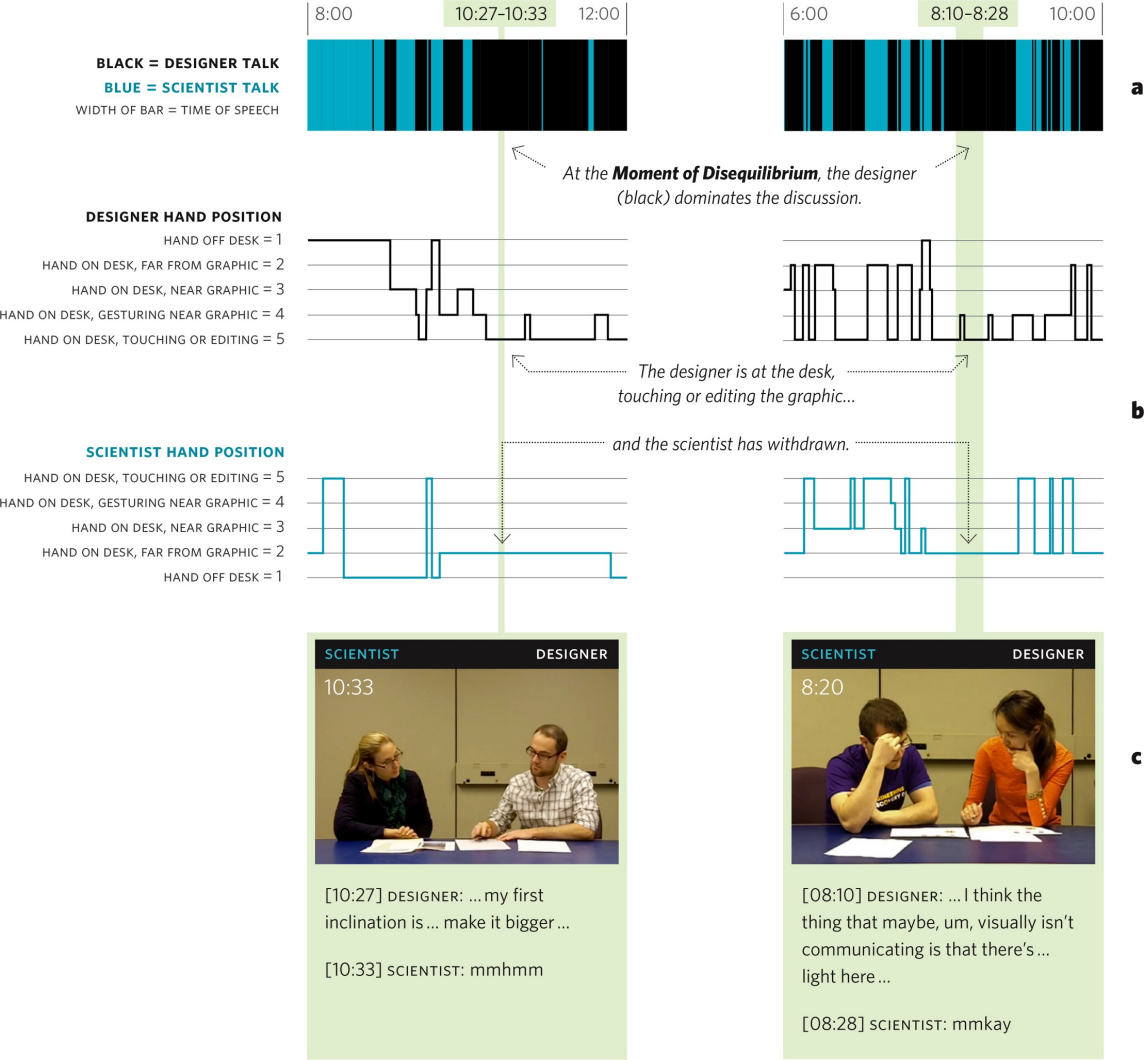
A closer look at the ‘moment of disequilibrium’. Two instances of disequilibrium characterized by (a) who is speaking, (b) hand position, and (c) body language. In (a) designer and scientist utterances/turn-taking are shown in a bar chart. In (b) hand positions are mapped, with higher numbers indicating closer proximity to the desk and graphic. Both (a) and (b) show the scientist withdrawing verbally and physically during disequilibrium. In (c) both scientists exhibit the discomfort of disequilibrium (clasped hands and crossed arms are known signals of stress).

In all twelve sessions, the scientist demonstrated a visible and audible ‘Moment of Disequilibrium’ when they realized that the graphic might need a substantial alteration of a kind that they had not imagined before—perhaps even a major rethinking and/or restructuring of the design. As shown in Fig 3, the signatures of *Disequilibrium* in dialogue, hand position, and facial expressions were readily detectable and pronounced.[39]

During the ‘Moment of Disequilibrium’ (and this was confirmed in every Design Help Desk session) the scientist expressed less words—typically, monosyllabic utterances—or lapsed into extended silence. As noted by Sacks et al, when silences occur at normal turn-taking junctures, this is usually a sign that the dyadic interaction has faltered.[40]

Our interpretation of *Disequilibrium* is further reinforced by the body language and gestures of scientists. Physically, the scientists withdrew their body and hands from the shared desk and paper. As noted by Graesser in his study of learner postures, the position of the body often changes when a learner experiences the confusion and frustration associated with cognitive disequilibrium. Crossed arms are also suggestive of a subject’s defensive stance. Pease describes this posture as a “barrier signal”—an unconscious attempt to block out a threat or undesirable circumstance.[41] Similarly, a subject with clasped (or fidgety) hands—as occurred during *disequilibrium—*indicates discomfort, nervous tension, and/or fear. Similar learning signatures (accompanied by these kinds of moments of disquiet) were evident in a classic study involving incumbent engineers at an aerospace workplace when two methodologies were compared for active engagement, deep understanding and near and far transfer.[42] We can also examine the dialogue that occurs during the moment of Disequilibrium:

**Designer:** Okay. So we've got (2.0) just look at (2.0) I'll do a draw…I'll leave this as like the original (Hhhh) um.. two ((inaudible)) things here
**Scientist**: Mmhmm
**Designer**: okay (Hhhh) um (2.0) 'cause what we would normally say (2.7) is like (2.0) you know (2.0) if this is sort of (Hhhh) I would say these floating numbers are sort of the issue that I see (Hhhh)
**Scientist:** Yeah I was trying to bring them outside of the box.. but for me it's still (hh) like.. I feel like each one should probably still be labeled
**Designer**: Yeah (2.3) um (2.3) and even.. you know (Hhh) you're giving it sort of an x and y
**Scientist**: Mmhmm

As evidenced in this excerpt, the moment of *Disequilibrium* appears when the designer states: “…these floating numbers are sort of the issue.” The scientist first reacts with what could be construed as a defense, by saying “I was trying…” but trails off in his effort to argue this point.

**Fig 4:**
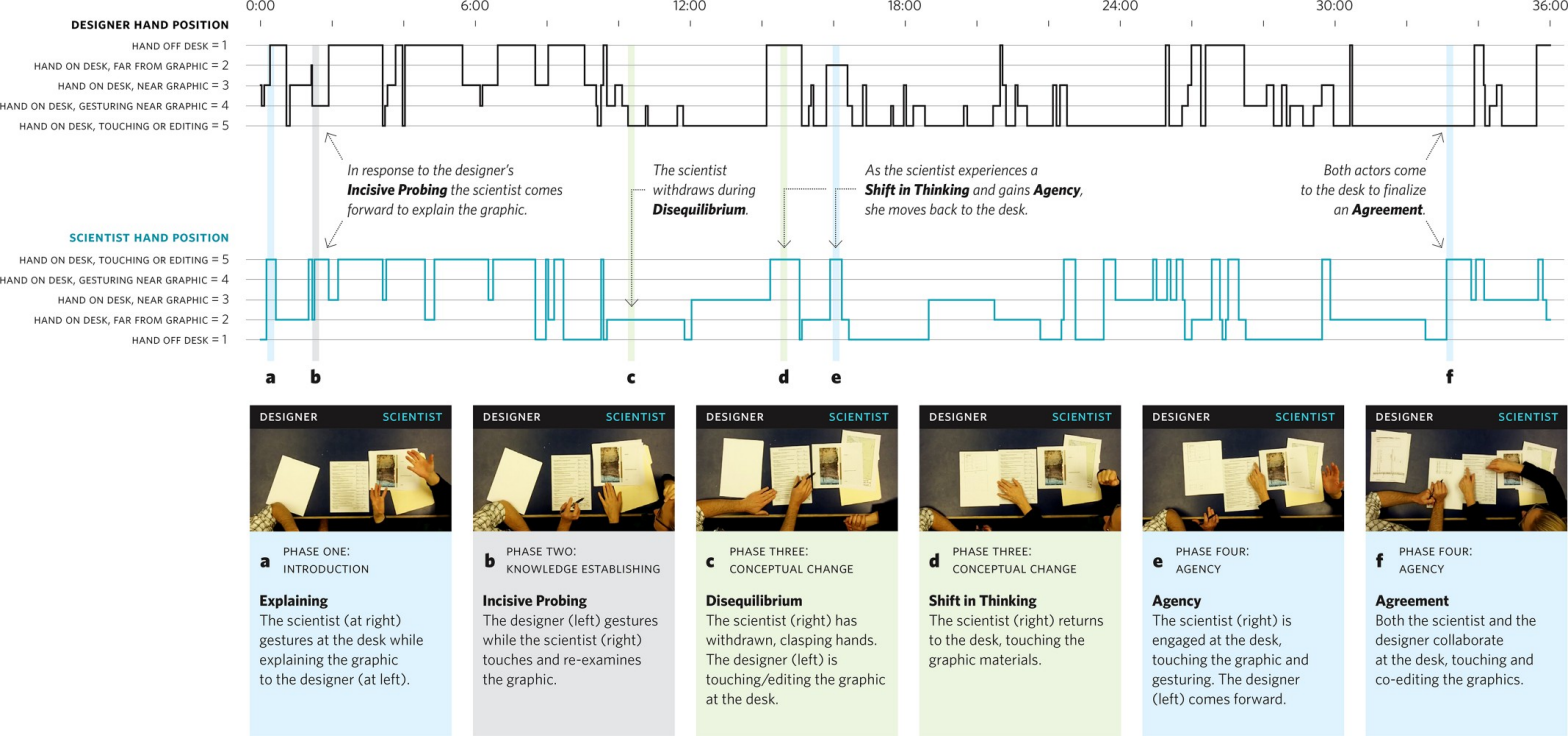
Mapping the interaction between designer and scientist through hand position. We plot the hand position of both the designer (left) and the scientist (right) as captured with an overhead camera at the Design Help Desk. Higher numbers indicate closer proximity to the visual (the graphic). Video stills illustrate the specific phases of a Design Help Desk session as described in Fig 1.

As seen in Fig 4A, the body language and gestures at the ‘Moment of Disequilibrium’ are in direct contrast to the beginning (and indeed the end when generating new ideas and contributing solutions) of a typical session, when the scientist was close to the desk and used hand gestures to *Explain* his or her thinking as part of the *Introduction* phase.

Similarly, the scientist’s behavior during the ‘Moment of Disequilibrium’ also contrasts the *Knowledge Establishing* phase. In this phase, when the designer asked *Incisive Probing* questions (shown in Fig 4B), the scientists leaned in, placing their hands on the desk and touching the graphic. Often, after the designer asked a question, he or she would pull away from the table in an open posture (hands in view, uncrossed limbs, clear eye contact) in order to “make space” for the scientist’s explanations. According to Pease, the designer’s body language invites the scientist to enter the designer’s space.

Given the silence from the scientist during *Disequilibrium*, the designer dominates the utterances during the *Conceptual Change* phase (184 vs. 11). The designer, who is aware of the scientist’s discomfort, tries to quickly shore-up the apparent ‘reactive’ response and makes attempts to invite the scientist into a more ‘generative’ space where they will be able to accept the situation, and offer meaningful solutions that can be articulated for consideration. For example:

**Designer**: Um (0.2) well it's a good start. You're starting to establish a series by carrying these symbols over (0.2) and then the next thing would be to bring these symbols over to the molecules as well (.2) so like,(0.3) maybe next to the A (0.2) there's like a little white triangle or whatever symbol corresponds to this molecule (0.3) so (0.2) that's just another way to tie this into the series (Hhhh) um (0.3) For (0.2) for this one (0.2),you said it's maybe not so important that you can't really read this data? Like the (0.3) the more important thing you get out of it is that they're all kind of r- in the same range (0.2) or like that they're all in on top of each other? And then (0.2) and then this is the most important part that they be able to read?
**Scientist:** Yeah (0.2) this is the most important part =
**Designer: =** 'Cause that's coming through pretty nicely =
**Scientist:** = Yeah =
**Designer**: = Like you can distinguish the lines (0.2)
**Scientist**: Mmhmm

In learning sciences pedagogical parlance, this is akin to adjusting the internal cognitive state of a learner by moving him/her out of the amygdala (reactive) brain by accessing the ‘generative’ executive function brain of the prefrontal cortex for deep engagement in a learning space.[43, 44]

After the ‘Moment of Disequilbrium,’ the scientist (who through nonverbal and verbal behaviors had withdrawn figuratively and literally from the joint attentional space on the desk, as seen in Fig 4C) re-emerges and re-engages with the designer. We see this re-engagement as evidence of a *Shift in Thinking*—the end of the *Conceptual Change* phase. The scientist tentatively begins to return to the desk and papers, seeking to understand the designer’s input by evaluating, consolidating, and synthesizing information and suggestions.[45] For example:

**Scientist**: So If I'm (0.2) if I'm trying to group (0.2) group this guy (0.1) this guy(0.2) this guy (0.2) and let's say, (0.2) like (0.2) these guys (0.2) all into one big (0.2) you know (0.3) they're all kind of (0.2) I mean they're,(0.2) they're different minerals (0.2) like (0.2) they say different stuff next to 'em (0.2) but they're all supposed to indicate more water (0.2) if I did (0.3) like (0.2) different shades of blue (0.3) for all of these (0.3) instead of yellow and red and brown and ?
**Designer:** Mmhmm ((nodding))
**Scientist:** Like (0.2) s-things like that (0.3) do you think that would (0.2) is-is that what you were kind of talking about?
**Designer:** Yeah

Similarly, when the scientist *Shifts in Thinking*, their hands come back to the graphic (as seen in Fig 4D) indicating an acceptance for the designer’s suggestions and a desire to seek a way forward. The designer again pulls back from the desk in order to give space to the scientist to generate new ideas. The scientist, newly engaged, points out specific parts of the graphic while alternative representations are proposed. The body language of the scientist confirms an active engagement (as seen in Fig 4E) leading into the final stage, *Agency*. We define Agency in a way that captures Scardamalia and Bereiter’s definition of ‘Adaptive Expertise’[46] in their groundbreaking work Surpassing Ourselves[47] thinking about it broadly as when a learner takes ownership for his/her own learning.

Towards the end of the session there is a renewed joint endeavor as both scientist and designer strive to finalize the project. The physical proximity of both parties, as shown in Fig 3F—something that has not occurred in the previous three phases—indicates a direct collaborative interaction between the two participants and is borne out by the discourse analysis (Fig 2).

As the allotted (~30 minute) Design Help Desk time winds down, the scientist begins *Taking Charge* and articulates a mutual *Agreement* that makes sense given the practical considerations for the graphic and their personal capacity for change. When the scientist determines the new direction and *Takes Charge*, their utterances once again surpass the designer (195 vs. 107). For example:

**Scientist**: Cool (0.2) So (0.2) so yeah (0.3) so on this one just kind of (0.2) essentially just take out all color out of text (Hhhh) is what you're saying on this one (0.2) like (0.2) just (0.3) ch-,er (hhh) change it (0.3) I mean (0.2) not all color (0.2) but (0.3) group these (0.2) in terms of blues (0.3) and (hhh) other ones in terms of black?
**Designer**: Mmhmm =
**Scientist**: = but (0.2) but too many colors happening on here?
**Designer**: yeah (0.2) too many colors happening (0.2) uh (0.3)
**Scientist**: yep

In addition to this interaction analysis, our results include the post-session satisfaction survey, which was completed by eight of the twelve participating scientists (~67%). This completion rate fits within norms, given the range described by Chapman and Jones in their study of response rates for online end-of-course evaluations.[48]

In the survey, the eight participating scientists chose either “agree” or “strongly agree” for the following three statements: 1) Overall the help desk consulting session was helpful to me; 2) The help desk consultant was knowledgeable and analytical; 3) The help desk consultant provided helpful direction and feedback. For the fourth statement (The help desk consultant established rapport with me) one scientist selected a neutral response (“neither agree nor disagree”) while the other seven selected either “agree” or “strongly agree.”

When asked what aspects of the session contributed most to their learning, two scientists noted that “getting an outsider’s perspective” was particularly helpful. One scientist also appreciated getting “straight forward, to the point and very helpful advice”—while another said it helped to get “clear and precise ways to change my figure—i.e., changing colors, aligning labels, etc.”

One scientist summed up her experience by noting, “I really liked how the consultant first tried to understand our project and then gave us helpful advice on how to organize the layout of the poster.”

When asked if anything detracted from their experience at the Design Help Desk, two scientists made small suggestions. One person felt that the cameras were distracting (in this case, the operator had an unusual difficulty with the overhead camera and caused a delay). Another scientist thought a visual designer who knew more science might be a better consultant. However, two others noted that a key advantage to the Design Help Desk was “getting a non-scientific perspective of my work.”

In our later follow-up with participants, we were able to determine the trajectory of all graphics after the Design Help Session. Eight of the twelve participants published their Design Help Desk graphic in an academic paper. Two of remaining participants had brought poster designs to the Design Help Desk; one poster was presented at a scientific conference and the other was shown in a public presentation at the end of a capstone course. One participant published her graphic in her graduate thesis. The final participant encountered a difficulty with her research that resulted in a significant change to her study; for this reason, her graphic was placed on “indefinite hold.”

**Fig 5:**
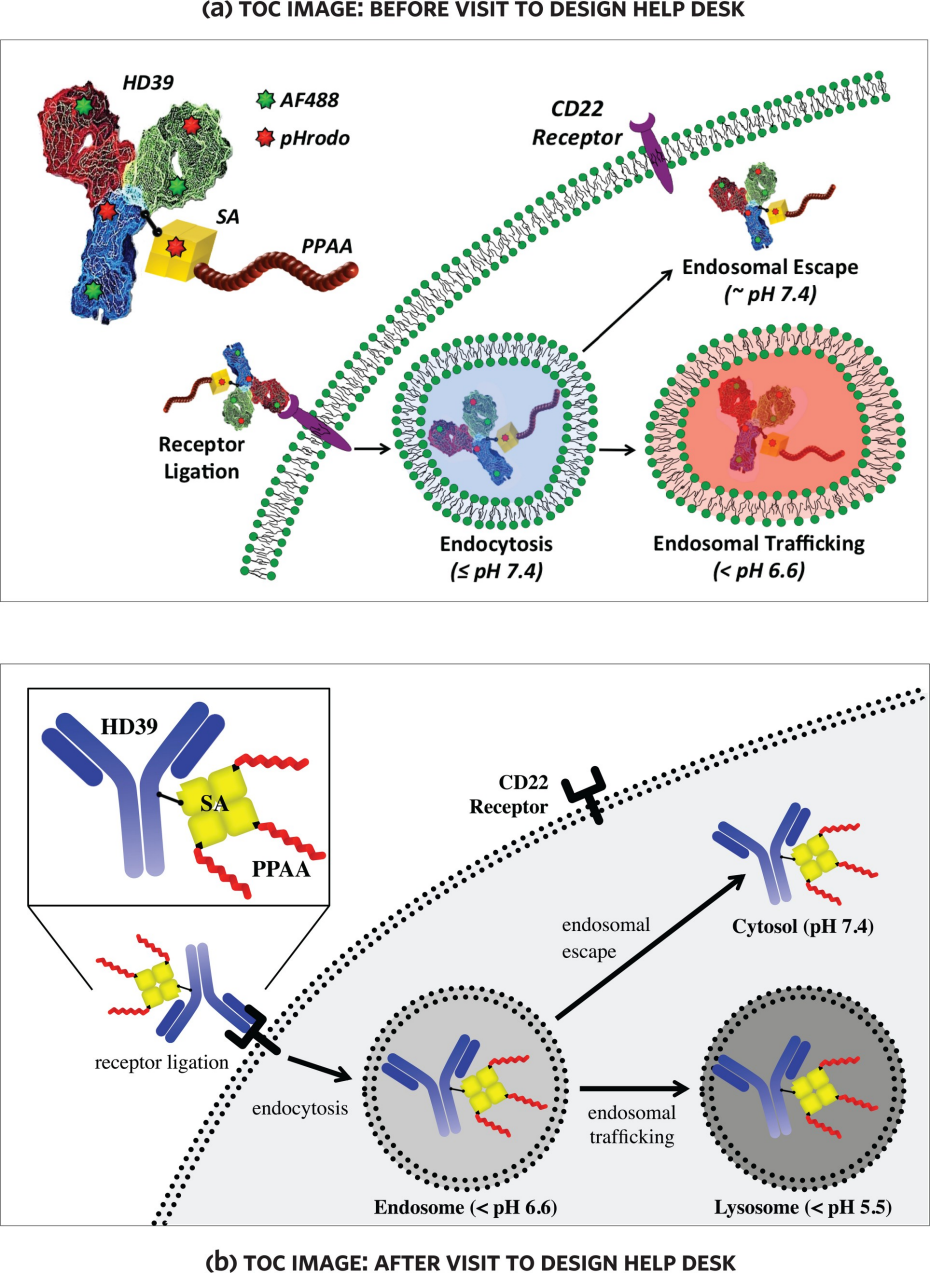
An improved figure before and after consultation at the design help desk. **Lower image from “**Intracellular Delivery and Trafficking Dynamics of a Lymphoma-Targeting Antibody–Polymer Conjugate”[49].

Of the eleven graphics that were published, six demonstrated clear improvements to the visual design—for example, simplification of overly complex illustrations, or reorganization into simpler compositions (reading top-to-bottom or left-to-right) Fig 5 (below) is typical of this kind of transformation.

Of the remaining five graphics, three exhibited minor changes after the DHD session (for example, small changes in color or better subtle repositioning of elements). The last two graphics were essentially unchanged, because those DHD sessions focused on reproduction and technical/software issues that did not involve changes to visual design.

Notably, during our follow-up effort, several scientists expressed appreciation for the Design Help Desk service. One scientist noted, “I found the Design Help Desk to be very helpful because it shifted the way I was looking … I think as students we tend to try to emulate figures we have seen in publication, whether or not they were particularly effective. Having someone outside my field, with expertise in visual communication and design, helped me learn to ‘see’ a figure I’m working on and ask what the goal of the figure is, and ask whether it can be understood, in a general sense, by anyone.” Another said, “It was especially nice to be able to discuss [my graphic] with a non-colleague [because] my peer-set at the time was mostly like-minded people pursuing similar fields. My external school/research friends would agree with whatever I said about my work, so it was extremely helpful to have an individual dedicated to hearing me out and providing useful feedback.”

Two of the scientists shared their published work with us prior to our scheduled follow-up simply to express thanks and gratitude for the service. One of the scientists was satisfied enough to list the Design Help Desk in the acknowledgments of his papers.[50, 51] In our follow-up, he wrote, “I can see that the final graphics were significantly improved compared to those that I brought in—the text was decluttered and made into a uniform style; the schematic drawings were made clearer by depicting what was happening below the surface; and the titles [are now] more descriptive and illuminating…much better figures through the help of your team.”

## Discussion

We see from post-session surveys and subsequent follow-up contact that scientists who came to the Design Help Desk were satisfied with their experience. We also see that scientists specifically used the service to improve graphics that were targeted for publication and distribution to the scientific community and general public. By examining the graphics before-and-after their visit to the DHD, we see that design consultations have the potential to greatly improve the quality of scientific visuals. While not all graphics were improved to the same degree, even small revisions have potential to enhance scientific communication and understanding.

Furthermore, in this study, we offer direct evidence from participant discourse and nonverbal communication to verify that peer interaction at the Design Help Desk helps scientists to learn visual design and, consequently, should help improve their scientific communications.

Specifically, at the Design Help Desk, scientists experienced conceptual change through a collaborative process that promotes learner engagement and agency. In response to the designer’s *Incisive Probing*, the scientist re-evaluates the visual design choices that s/he made previously. We hear the scientist second-guess choices they made when creating their original graphic, and see them experience *Disequilibrium*, as evidenced by extended silences, monosyllabic utterances, and physical withdrawal from the shared space.

Through our twelve cases, we see that the Design Help Desk intervention had a meaningful impact on scientists, with *Disequilibrium* appearing to be the catalyst. A design-based interaction between a visual designer and a scientist highlights and ‘makes visible’ a *Disequilibrium* that (in a pedagogic world) galvanizes learning. The learning is characterized by *Conceptual Change* [52] during an intentional process where the scientist’s prior knowledge is first ‘made visible’ and then unsettled.

What factors made the Design Help Desk successful? First, the unique nature of the participants had a meaningful impact—two experts who were also intelligent novices. Because the designer is not familiar with the scientist’s work, the scientist is forced to explain his visualization. Many researchers have shown that controlled verbalization can facilitate learning.[53] As described by Ahlum-Heath and diVesta the quality and timing of verbalization events (rather than the mere activity of verbalizing) contribute to the outcome in either a positive or negative way. Intentional focus on the process of Explanation at the Design Help Desk (e.g., the designer’s *Incisive Probing)* compels the scientist to confront “blind spots”[54] that might have been hidden in his understanding of the visual design problem.

Second, sessions at the Design Help Desk were short episodes (approximately 30 minutes), which dealt with a constrained problem—a single scientific graphic. Research confirms that sharing focus on an object of ‘joint visual attention’ helps individuals communicate during an interactive session and fosters the social engagement that significantly predicts learning in areas related to the content of the common space.[55, 56] Additionally, a 30-minute interval is often referenced by productivity experts as an ideal length for a work session[57]; the time pressure enhances attention and focus on the largest opportunity or problem area.[58]

Finally, the Design Help Desk represented a “safe space” where scientists could find their voice,[59] generate new ideas,[60] and come away with unique, adaptive solutions.[61] There were no external rewards or punishments at the Design Help Desk. Scientists were drawn to the service by an intrinsic[62] desire to improve their visualizations. Similarly, the design consultants agreed to work at the Design Help Desk due to their interest in teaching others[63] about design. The self-selected nature of both participants likely smoothed the way toward a positive outcome,[64] where designers were willing to grapple with a complex, technical subject, and scientists were able to change course and reinvest in a new approach. In ordinary life situations, disequilibrium may result in social withdrawal in a protective stance,[65] but at the Design Help Desk, the designer was able to engage with the scientist, help them “let go” of preconceptions, and spur learner agency.[66]

## Conclusion

This study examined what happened during interactions between a designer and scientist at the Design Help Desk, a free tutoring service offering visual advice in a one-on-one setting to scientists and engineers who sought to improve their visuals and data visualizations in publications, posters and presentations. Twelve consultation sessions were analyzed. All sessions involved a single scientist or engineer (a graduate student or post-doctoral researcher studying science or engineering at the University of Washington), and a designer (a senior undergraduate or a graduate student studying design at the University of Washington). The purpose of this research was to understand what kind of learning was taking place between scientists and designers at the Design Help Desk, and to determine if design-science consultations are a viable means of helping scientists and engineers acquire visual design skills.

At the Design Help Desk we observed active learning during all sessions/interactions between a scientist and a designer. During all twelve sessions, scientists overcome misconceptions and a fixed mindset[67] by negotiating a moment of *Disequilibrium* that facilitated *Agency* and understanding. The sessions satisfied the scientists and improved their visuals. The success of the Design Help Desk intervention supports the notion that an interaction between STEM (Science, Technology, Engineering and Math) and the Arts (by pairing design and scientific thinkers in STEAM programs) promotes not only better science,[68, 69] but indeed, better learning.[70–72]

The idea of bringing designers and scientists together has, in the past, proven to be successful in education. In 2007–2010, the National Science Foundation sponsored a series of “Picturing to Learn” workshops that connected science students and faculty from Harvard, MIT, Duke University and Roxbury Community College with design students and faculty from the School of Visual Arts in New York City.[73] As noted by scientist and organizer Felice Frankel, “When science and design students collaborate, their drive to understand one another’s ideas pushes them to create new ways of seeing science.”[74]

We hope that findings from our research that examined interactions between designers and scientists within a learning sciences framework will enable the development of more cross-disciplinary peer tutoring centers in design modeled after the Design Help Desk. Such design/science centers would be relatively simple to setup and replicate, given that there are ~2,500 degree programs in or related to graphic/visual design in the United States[75] and tutoring only requires a relatively small (two-person) desk/discussion space (typically available at university libraries or other common study areas). Students from design programs are often eager to share their knowledge with others—as volunteers, or for academic credit, or for a modest hourly wage. Additional Design Help Desks could help nurture better-quality visualizations and graphics in science and encourage cross-fertilization of ideas and future collaboration between the scientific, learning sciences, and visualization communities.

## Appendix A

### Design Help Desk Participant Survey

Thank you for participating in the Design Help Desk research project. The following questions will help us know a little more about you and your program, and how we can better help others like you to make better diagrams and graphics for their poster, paper and other academic presentations.

Q1.

Please enter the identifying code that you received in your email for this survey.

Your name will not be associated with your answers.

Q2.

My bachelor's degree is in:

(e.g. Chemistry with a minor in Biology)

Q3.

Home department:

Q4.

Current status in graduate program:

passed qualifying exam at UW

passed general exam at UW

passed final exam at UW

not in a graduate program; already have Ph.D.

Other:

Q5.

How many years have you been enrolled in a science or engineering graduate (post bachelors) program? Include years spent at UW and elsewhere, if applicable.

Enter a number (without commas).

Q6.

Rate how well the statement applies to your research:

My work contributes to nanotechnology research.

Strongly disagree

Disagree

Neutral

Agree

Strongly agree

Q7.

I have prepared graphics for: (check all that apply)

unpublished work: e.g. group meetings, advisor meetings

conference posters

conference presentations and/or public talks

papers for peer-review

Other:

Q8.

Do you have any formal training on how to make scientific graphics?

Check all that apply.

Single session lecture or seminar

Multi-session workshop

Quarter/semester long design course

No formal training

Other:

Q9.

When you create graphics, who are your sources for feedback? Select all that apply.

Advisor/faculty

Colleagues in your research group

Peers outside of your research group

Friends

Other:

Q10.

What has been the most useful influence on how you make scientific graphics?

Peer feedback

Advisor feedback

Formal training

Other:

Q11.

What types of software do you use in creating your scientific figures and graphs? Rate your proficiency in using each of these tools:

Microsoft Powerpoint: Power User

Microsoft Powerpoint: Good User

Microsoft Powerpoint: Average User

Microsoft Powerpoint: Weak User

Microsoft Powerpoint: Have Never Used

Microsoft Excel: Power User

Microsoft Excel: Good User

Microsoft Excel: Average User

Microsoft Excel: Weak User

Microsoft Excel: Have Never Used

Adobe Photoshop (or similar): Power User

Adobe Photoshop (or similar): Good User

Adobe Photoshop (or similar): Average User

Adobe Photoshop (or similar): Weak User

Adobe Photoshop (or similar): Have Never Used

Adobe Illustrator (or similar)

Adobe Illustrator (or similar): Power User

Adobe Illustrator (or similar): Good User

Adobe Illustrator (or similar): Average User

Adobe Illustrator (or similar): Weak User

Adobe Illustrator (or similar): Have Never Used

Publication quality graphing software (e.g. Origin, gnuplot, Matlab, Igor Pro, etc.): Power User

Publication quality graphing software (e.g. Origin, gnuplot, Matlab, Igor Pro, etc.): Good User

Publication quality graphing software (e.g. Origin, gnuplot, Matlab, Igor Pro, etc.): Average User

Publication quality graphing software (e.g. Origin, gnuplot, Matlab, Igor Pro, etc.): Weak User

Publication quality graphing software (e.g. Origin, gnuplot, Matlab, Igor Pro, etc.): Have Never Used

Other: Power User

Other: Good User

Other: Average User

Other: Weak User

Other: Have Never Used

Q12.

If you indicated so above, which publication quality plotting software do you use? Any other programs?

Q13.

Rate the importance of the following questions:

How important is the design of graphics in science?: Extremely Important

How important is the design of graphics in science?: Very Important

How important is the design of graphics in science?: Neutral

How important is the design of graphics in science?: Kind of Important

How important is the design of graphics in science?: Not important

How important is it to teach science students to make graphics?: Extremely Important

How important is it to teach science students to make graphics?: Very Important

How important is it to teach science students to make graphics?: Neutral

How important is it to teach science students to make graphics?: Kind of Important

How important is it to teach science students to make graphics?: Not important

Q14.

Number of sessions you have used the Help Desk before this session:

Enter an integer (without commas).

Q15.

Please rate your experience at the Design Help Desk:

Overall, the help desk consulting session was helpful to me: Strongly disagree

Overall, the help desk consulting session was helpful to me: Disagree

Overall, the help desk consulting session was helpful to me: Neither agree nor disagree

Overall, the help desk consulting session was helpful to me: Agree

Overall, the help desk consulting session was helpful to me: Strongly agree

The help desk consultant was knowledgeable and analytical: Strongly disagree

The help desk consultant was knowledgeable and analytical: Disagree

The help desk consultant was knowledgeable and analytical: Neither agree nor disagree

The help desk consultant was knowledgeable and analytical: Agree

The help desk consultant was knowledgeable and analytical: Strongly agree

The help desk consultant established rapport with me: Strongly disagree

The help desk consultant established rapport with me: Disagree

The help desk consultant established rapport with me: Neither agree nor disagree

The help desk consultant established rapport with me: Agree

The help desk consultant established rapport with me: Strongly agree

The help desk consultant provided helpful direction and feedback: Strongly disagree

The help desk consultant provided helpful direction and feedback: Disagree

The help desk consultant provided helpful direction and feedback: Neither agree nor disagree

The help desk consultant provided helpful direction and feedback: Agree

The help desk consultant provided helpful direction and feedback: Strongly agree

Q16.

What aspects of the help session contributed most to your learning?

Q17.

What aspects of the help session detracted from your learning?

That's it! Thanks again for your participation and feedback.

Questions or Comments?

Contact Timothy OMahony at tko2@u.washington.edu

## Appendix B

### Transcription Conventions

(?) talk too obscure to transcribe.

Hhhhh audible out-breath

.hhh in-breath

= overlapping talk

(.) silence, less than half a second

(..) silence, less than one second

(2.8) silence measured in 10^ths^ of a second

:::: lengthening of a sound

Becau- cut off, interruption of a sound

he says. Emphasis

= no silence at all between sounds

LOUD sounds

? rising intonation

(left hand on neck) body conduct

[notes, comments]

## Appendix C

### Design Help Desk Consultant Script

The Design Help Desk research seeks to investigate and understand how scientists learn about visual communication (graphics and diagrams for research papers and presentations) at the tutoring center. As part of this team of researchers, consultants with a Visual Design and Communications background work with non-visual design scientists in order to understand this creative space. The following script is suggested in order to facilitate a productive face-to-face meeting with each scientist concerning their design graphic, and to insure a positive, collaborative and pleasant experience for all concerned. We have divided the script into two formal sections to distinguish the process from the product.

#### PROCESS

The process involves the physical location, the interaction and the tone of the discussion with the prospective candidate. Here are a number of suggestions that might make this experience pleasant and productive for the consultant and the candidate.

**Greeting**

- Consultant to meet the candidate in the foyer at CNT to escort him/her into the Help Desk studio
- Friendly greeting and welcoming attitude—create a safe learning environment
- Words that put the candidate at ease and secure in the knowledge that this is a place where learning takes place

#### PRODUCT

The product refers to the candidate’s graphic or diagram. Being mindful and respectful of the difficulties that some people might experience in showing an “outsider” their work, we offer the following suggestions.

**Listening**

- Begin with an overview–ask what the area of work is and what this particular graphic(s) represent in his/her own words
- Rules of operation–is it ok to mark up your graphics

**Know Your Audience**

- Is this graphic for a paper, a ppt or presentation
- How critical is the timeline
- Who are the stakeholders—co-authors, lab, etc.
- Be cognizant of the candidate’s level of engagement, fears or skills

**Offering Suggestions**

- Careful choice of words and timing help allay any fears of looking ridiculous or stupid
- Direct approach which presumes to improve the work might help
- Offer choices
- Praise what is working in the work and offer solid suggestions for what isn’t

**Wrap Up**

- It’s important to be courteous and helpful in the letting go
- Important not to discuss the work with people outside the researcher group

## Supporting information

S1 Fig (EPS)

S1 File (PDF)

S1 Video Depicting a typical interaction between designer and scientist at the design help desk. At the moment of disequilibrium, designer (always on the right side of frame) dominates interaction, scientist withdraws verbally and with body language
(MOV)

S2 Video Video depicting a typical interaction between designer and scientist at the design help desk. At the moment of disequilibrium, designer (always on the right side of frame) dominates interaction, scientist withdraws verbally and with body language
(MOV)
