# Supplementary material for: The Design Help Desk: A collaborative approach to design education for scientists and engineers
Source: PLoS One. 2019 May 1;14(5):e0212501. doi: 10.1371/journal.pone.0212501 (PMC6493711; doi:10.1371/journal.pone.0212501)
Supplement: S1 File — (PDF) [file pone.0212501.s002.pdf]

Supplementary Materials

The Design Help Desk: A Collaborative Approach to Education in Science and Engineering Visualization

T. K. O'Mahony[1, 2], J. Petz[3], J. Cook[4], K. Cheng[3], M. Rolandi[1,5]*

[1] Department of Material Science & Engineering, University of Washington, Seattle, WA

[2] LIFE (Learning in Informal and Formal Environments) Center, College of Education, University of Washington, Seattle, WA

[3] Visual Communication Design, Division of Design, School of Art, University of Washington, Seattle, WA

[4] Interaction Design, Division of Design, School of Art, University of Washington, Seattle, WA

[5]Department of Electrical Engineering, University of California, Santa Cruz, CA 95064

*Correspondence to: tko2@uw.edu, rolandi@ucsc.edu

Subjects and Methods

Subjects

Subjects for the Design Help Desk research project self-selected from the sciences population (graduate and postgraduate) of the University of Washington campus. This resulted in a cross-section of participants (n=16) who came with backgrounds in the physical sciences, theoretical sciences, and nano-sciences. For example, several subjects produced graphics that pertained to physical sciences (e.g., geologic and stratigraphic charts), including one individual, whose work centered on imagery from the planet Mars. While it was not a particular focus of our study we did note that there was an equal number of females to males in the candidate list and age of participants ranged from mid twenties through mid fifties. Similarly, we did not focus on diversity or ethnic background, but we note that sample participants came from diverse backgrounds reflecting the general population of the University of Washington campus. Participants learned about the Design Help Desk from a number of sources as described here (see appendices for more information). Sources included (i) advertisement brochures that were strategically placed on notice boards and residence halls for University of Washington science students; (ii) pre-lecture announcements by faculty and staff during the set-up phase of the project; and (iii) word of mouth across campus as people found out about the availability of a source of help in designing and assessing graphic and data representations for papers and publications. Subjects signed up via electronic service that guaranteed a time slot with a designer on a given day and time for 30 minutes. Participants were informed that they would be asked to participate in a design study (this information was clearly written on the advertisements and during announcements in classrooms). Internal Review Board permission was sought and it was

granted for this study. All necessary precautions were taken to preserve participant anonymity and express written permission was sought and granted when pictorial images and verbal utterances of participants were used in associated publications and reports.

When subjects arrived at the Design Help Desk, they were reminded that the interaction was part of a design experiment and asked to sign a consent form. Subjects were informed of their rights and told that if they preferred not to take part in the experiment that it would have no impact on whether or not the designer would help them. In fact the designer never knew if a participant was in the study or not. As it happened all participants opted to be part of the study. The setup at the Design Help Desk was always the same. The designer (on the right facing the camera) and scientist (on the left facing the camera) sat at the same desk facing forward directly ahead into the video camera. Meanwhile, another video camera was placed directly over the desk (in the ceiling) and was focused on what happened on the desktop. Both cameras were turned on before the session begun and were not turned off until after the scientist left the room.

Data

Data comprised a mix of written and video/audio material. In all cases, written demographic survey material was collected that described the subjects. In addition, all sessions were captured in a two-camera set up as described above. Face-on video captured the interaction of the designer and scientist at the Design Help Desk as they negotiated the review and repositioning of the data and representational outcome of that data with regard to a paper or other presentation. Data was analyzed using a mixed methods combination that connected interactants' discourse, embodied actions and outcomes. Discourse consisted of turn-taking utterances by each of the participants within the framework of the Design Hel Desk session described earlier.

Content logs (*1*), which captured key moments of activity and discourse, were created from the videotapes to aid analysis. Two researchers used these logs and the video recordings to independently identify significant interactional episodes. Using standardized transcription conventions, content logs, and field notes, we reconstructed in writing what the participants said and did in relation to one another, preserving the temporal sequence of the interactions. Participant verbal interactions were transcribed and coded (Supplementary Table 1). Emergent categories and themes in relation to discourse content and participant engagement (through questions that stemmed from discussions and interactions) were documented. Verbal interactions were analyzed for sequences that captured participant meaning-making. We focused on two kinds of discourse analysis: (a) Using participant talk as a measure of interactivity in the Design Help Desk sessions, we coded and counted utterances to describe the reciprocity and types of interactions that took place; and (b) using discourse analysis from a grounded theory perspective (*2, 3*), we traced the emergence of patterns of interactions over the session discussions.

In order to process large amounts of discourse data and carry out a conversation analysis (particularly to make it accessible in a visual landscape), we utilized a custom software application that produced visual graphs of the interactants' conversations. To be effective communicators, humans must appropriately encode, decode, and regulate nonverbal cues, such as speech rate, pitch, facial expression, and body language. This software captures nonverbal cues in real time and displays visual feedback (*4*), allowing us to visualize scientist and designer engagement at the Design Help Desk. The software algorithms facilitated an analysis of transcripts for conversation patterns that 'made visible' predefined aspects of participant interaction, highlighting turn-taking, over-talk, and argumentation. This method is grounded in a literature on human computer interface that investigates exchange of ideas in conversation as well as

non-verbal cues in specific communication situations (*5*). In this emergent field where human computer factors are studied, scholars investigate oral traditions where a typical residue (*6*) of written language is augmented with discursive interaction that is valued from gestures and body-language (*7*). By visualizing the content logs of conversations (*8*) researchers are able to take into account volume, pitch and content so that artifacts are codified into meaningful social digestion

STAGE	CODE	DEFINITION	EXAMPLES	UTTERANCES (average)
**ONE Introduction**	**Making Visible**	Describes the designer helping the client see the work/graphic from a new perspective.	[02:32] CLIENT: I'm actually like, capturing the, the same stuff on both of the images, uhm, *((0.3 second delay))*, so, that's, that's kind of the point of this figure.	17
	**Asking Permission**	Describes a situation where the designer asks the client for permission to edit or make changes to the client's drawing.	[01:06] DESIGNER: Do you mind if I mark it up (referring to the copy of his drawing)	3
**TWO Knowledge Establishing**	**Explaining**	Describes the client's response to the designer when he/she is asked to describe the graphical representation of the work in question.	[02:28] CLIENT: So, we are looking at sort of three different conditions here ... so, this is what we call laminar flow, which is this section, and that is when a fluid is very fluid and steady ... ahmmm	12
	**Incisive Probing**	Describes a condition where the designer asks probing questions to help make visible what the client is representing.	[02:10] DESIGNER: So your Figure 1... this is actually ... ahmmmm you can see that it is ... like this is ahmm its not birds eye view ... you're not looking at it straight on, right?	6
**THREE Change**	**Disequilibrium**	Describes how people come to learn by experiencing discrepancies between what they already know and what they discover in their environment.	[03:43] DESIGNER: So all these ... these correspond CLIENT: _Right DESIGNER: = ok. Ahmm... I didn't get that initially...	2
	**Shift in Thinking**	Refers to the client's changed understanding of a concept or idea that emerges from the discussion about the graphic in question.	[03:44] CLIENT: = and it sort of dominates the image ... I don't think this is like, obvious enough to be on the side or ... but if ...	4
**FOUR Knowledge Sharing**	**Agreement**	Reflects a collaborative examination of the graphic where both actors agree that they understand the problem and have a working solution.	[05:46] CLIENT: = one sided yeah DESIGNER: = yeah CLIENT: = ok DESIGNER: yeah, that will increase the effects	10
**FIVE Closing**	**Overtalk-Confirmatory**	This code (denoted with the equal sign "=") refers to when one of the dyad interrupts the other, usually either in enthusiastic confirmation or in quick disagreement.	[05:13] DESIGNER: And so this is CLIENT: = yeah this is... DESIGNER: = the same deal CLIENT: = the same deal, yeah	17
Throughout	**Joint Visual Attention**	Corresponds to both actors focusing their attention, words and embodied actions on a common object at the same time.	*This aspect has to be viewed on-camera to verify*	12

Supplementary Table 1. Cognitive Interaction Coding. Typical Design Help desk session surfaced identifiable stages (described in column 1) in the interactive dialogue between designer and scientist. Stages included Introduction, Knowledge Establishing, Change, Knowledge Sharing and Closing. Various codes were associated with these stages in the dialogue and are described in column 2 (naming), column 3 (description), and column 4

(example). The final column - Utterances – correlates the average amount of speech over time in each of the categories that are coded. Note that the most critical category code, Equilibrium, embodies the least utterances.

Design Help Desk Consultant Script

The Design Help Desk research seeks to investigate and understand how scientists come to construct and present graphics and diagrams for research papers and presentations. As part of this team of researchers, consultants with a Visual Design and Communications background work with non-visual design scientists in order to understand this creative space. The following script is suggested in order to facilitate a productive face-to-face meeting with each scientist concerning their design graphic, and to insure a positive, collaborative and pleasant experience for all concerned. We have divided the script into two formal sections to distinguish the process from the product.

Process:

The process involves the physical location, the interaction and the tone of the discussion with the prospective candidate. Here are a number of suggestions that might make this experience pleasant and productive for the consultant and the candidate.

Greeting

- Consultant to meet the candidate in the foyer at CNT to escort him/her into the Help desk studio
- Friendly greeting and welcoming attitude – create a safe learning environment

- Words that put the candidate at ease and secure in the knowledge that this is a place where learning takes place

Product:

The product refers to the candidate's graphic or diagram. Being mindful and respectful of the difficulties that some people might experience in showing an "outsider" their work, we offer the following suggestions.

Listening

- Begin with an overview – ask what the area of work is and what this particular graphic(s) represent in his/her own words
- Rules of operation – is it ok to mark up your graphics

Know your Audience

- Is this graphic for a paper, a ppt or presentation
- How critical is the timeline
- Who are the stakeholders – co-authors, lab, etc.
- Be cognizant of the candidate's level of engagement, fears or skills

Offering Suggestions

- Careful choice of words and timing help allay any fears of looking ridiculous or stupid
- Direct approach which presumes to improve the work might help
- Offer choices
- Praise what is working in the work and offer solid suggestions for what isn't

Wrap Up

- It's important to be courteous and helpful in the letting go
- Important not to discuss the work with people outside the researcher group

Appendix 2

Design Help Desk Advertisement Flier

DESIGN

HELP DESK

The Design Help Desk offers free advice to science students seeking to improve their publication figures and visuals.
Get help on your posters, illustrations, diagrams, data plots, slides, etc., while participating in a NSF-funded study.

THURSDAYS 1:00-4:00PM

Sign up online: http://tinyURL.com/DesignHelpDesk

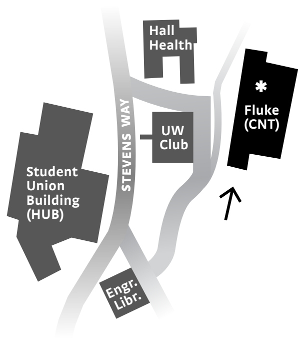

UW FLUKE HALL

Center for Nanotechnology (CNT)

References

1. B. Jordan, A. Henderson, Interaction Analysis: Foundations and Practice. *The Journal of the Learning Sciences* **4**, 39 (1995).
2. H. S. Becker, in *Ethnography and Human Development: Context and Meaning in Social Inquiry,* R. Jessor, A. Colby, R. A. Shweder, Eds. (University of Chicago Press, Chicago, 1995), pp. 53-72.
3. A. Strauss, J. Corbin, Eds., *Grounded theory in practice*, (Sage Publications, Thousand Oaks, CA, 1997).
4. R. A. Patel *et al.*, Leveraging visual feedback from social signal processing to enhance clinician's nonverbal skills. *Human Factors in Computing Systems*, 421 (2013).
5. T. Bergstrom, K. Karahalios, Social mirrors as social signals: Transforming audio into graphics. *Computer Graphics and Applications, IEEE* **29**, 22 (2009).
6. W. Ong, *Orality and literacy: The technologizing of the word*. (Routledge, New York, 2002).
7. P. Ekman, *Emotions revealed: Recognizing faces and feelings to improve communication and emotional life*. (Henry Holt & Co., New York, 2003).
8. P. Mathur, K. Karahalios, Visualizing remote voice conversations. *Human Factors in Computing Systems* **Extended Abstracts**, 4675 (2009).
